# Tiaojing Cuyun Recipe Enhances Pregnancy Outcome via the VEGF/PI3K/AKT/eNOS Signaling Pathway in EID Mice

**DOI:** 10.1155/2022/9461444

**Published:** 2022-02-24

**Authors:** Hongli Huang, Lei Xia, Yanqiu Xia, Yunping Yan, Zhuojun Jiang, Pei Zhao, Li Dong

**Affiliations:** ^1^Yueyang Hospital of Integrative Traditional Chinese and Western Medicine, Shanghai University of Traditional Chinese Medicine, China; ^2^The Public Experiment Platform, Shanghai University of Traditional Chinese Medicine, China

## Abstract

**Purpose:**

In this study, we evaluated the effect of Tiaojing Cuyun Recipe (TJCYR) on embryo implantation dysfunction- (EID-) induced damage of endometrial receptivity in mice and investigated the mechanisms underlying the effect.

**Methods:**

The main compounds of TJCYR were identified by high-performance liquid chromatography (HPLC). One hundred and twenty pregnant mice were randomly divided into six groups: control, EID only, progesterone (Prog)+EID, TJCYR-low-dose+EID, TJCYR-medium-dose+EID, and TJCYR-high-dose+EID. Mifepristone was injected to make the EID model. On the fourth day of pregnancy, serum was obtained to analyze hormone level by radioimmunoassay, the uterus was collected to analyze morphology by hematoxylin and eosin (H&E) and scanning electron microscopy (SEM), and a combination of immunofluorescence and Western blot was used to identify the related proteins. On the eighth day of pregnancy, the mice were sacrificed and the number of uterus-implanted blastocysts was counted.

**Results:**

Treatment with TJCYR significantly improved the number of implanted sites, the number of well-developed pinopodes, and microvascular formation in the mice. Moreover, TJCYR significantly activated PI3K/Akt/eNOS signaling pathways to promote angiogenesis, resulting in significantly improved endometrial receptivity and fertility outcomes when compared to the model group.

**Conclusion:**

These findings demonstrate that TJCYR was able to protect embryo implantation of EID mice due to TJCYR-mediated improvement in endometrial receptivity by promoting endometrial angiogenesis.

## 1. Introduction

The incidence of infertility has been a global health problem among women [1]. While access to assisted reproductive technology (ART) has overcome the majority of infertility causes, success rates have stagnated at around 30% [2]. Successful pregnancy inevitably depends on high embryo quality and good endometrial receptivity. During pregnancy, the endometrium apical surface undergoes several morphological, molecular, and biochemical changes to provide a favorable environment for embryo implantation, thereby creating an effective maternal-fetal interaction [3]. Thus, endometrial receptivity is key to raising the pregnancy rate in women with infertility [4–6]. Attempts at focusing on improvement of endometrial receptivity have been made in recent years, such as enhancing microcirculation and trophoblast invasion [7]. During the reproductive processes, the supply of essential nutrients and oxygen from vasculature is very important for the development of maternal-fetal interaction. It has been reported that the endometrium thickens with mature vascular network and increased blood flow, which reflect sufficient endometrial receptivity and, in particular, determine the endometrial response to the blastocyst at the early stage of embryo implantation [8]. Thus, enhanced endometrial angiogenesis is one of the important biological events during the reproductive cycle and pregnancy.

Traditional Chinese Medicine has gained wide acceptance due to its advantages of multifactorial and multitarget actions. In our clinical practice, we found that Tiaojing Cuyun Recipe (TJCYR) clearly enhanced the rate of pregnancy in women with infertility. Some studies reported that TJCYR may regulate follicular development and elevate the pregnancy rate in anovulatory infertility patients with kidney deficiency syndrome and significantly improve the ovulation rate and the reproductive function of the endocrine axis in androgen-sterilized rats [9, 10]. It has been reported that Bushen Huoxue Formula, which is composed of TJCYR, can improve the ACT-INH-FS system in patients with polycystic ovary syndrome with kidney deficiency and blood stasis [11]. Experimental studies have also demonstrated that *Epimedium brevicornu* Maxim. and *Morinda officinalis* How. improve endometrial receptivity in ovulation stimulation (OS) in EID mice through significant improvements in the spatial and temporal expression of pinopodes, accompanied by a significantly increased number of embryonic implantation sites [12]. These effects are desirable and satisfactory, but the therapeutic mechanism of TJCYR is not yet fully elucidated. The aim of this study was to provide a detailed account of the beneficial effect of TJCYR on EID-induced damage to endometrial receptivity in mice and to investigate its mechanism of action. From this perspective, we attempted to find novel candidates from traditional medicinal herbs to enhance the pregnancy outcome.

## 2. Materials and Methods

### 2.1. Medicine Preparation

TJCYR consists of seven herbs (Table 1). All herbs were supplied by the Shanghai Kangqiao Chinese Medicine Tablet Co., Ltd. (Shanghai, China). The decoction component mixture was added with water of 4 times volume, boiled twice, concentrated using a rotary evaporator, and attained the equivalent crude content of 2.0 g/mL. The concentrations of 12 g/kg, 24 g/kg, and 48 g/kg of the preparations were designated as TJCYR-L, TJCYR-M, and TJCYR-H, respectively.

### 2.2. Identification of Chemical Constituents in TJCYR by High-Performance Liquid Chromatography (HPLC)

The active ingredients of TJCYR were examined using the 1200 series HPLC device (Agilent Technologies, Santa Clara, CA, USA) with an autosampler (G1329B), thermostatted column compartment (G1316A), quaternary pump (G1311A), photodiode array detector (G1315D), and degasser (G1322A). HPLC was performed on an Apollo C18 (4.6 × 250 mm; particle size, 5 *μ*m; GRACE, Columbia, Maryland, USA) with a mobile phase of acetonitrile (A) -0.1% (*v*/*v*) and phosphoric acid (B) for gradient elution (0–70 min, 1-40% A; 70-90 min, 40-80% A). The detection wavelength was 260 nm, and the flow rate was 1 mL/min. The column temperature was 30°C, and the injection volume was 10 *μ*L. The standard solutions of Calycosin-7-glucoside, Acteoside, Salvianolic acid B, Icariin, Tanshinone IIA, and sample were filtered with a 0.45 *μ*m membrane filter before subjecting them to HPLC analysis.

### 2.3. Reagents

Mifepristone and progesterone were obtained from the pharmacy department of Yueyang Hospital of Integrative Traditional Chinese and Western Medicine; hematoxylin-eosin, Antifade Polyvinylpyrrolidone Mounting Medium, and nitric oxide (NO) assay kit were purchased from Beyotime (China); the antibodies ER*α*, ER*β*1, PR, Integrin *α*V, Integrin *β*3, OPN, LIF, eNOS, and p-eNOS were purchased from Abcam (UK); vascular endothelial growth factor (VEGF) and glyceraldehyde-3-phosphate dehydrogenase (GAPDH) were purchased from Proteintech (USA); Alexa Fluor 488 nm, Alexa Fluor 555 nm, Akt, p-Akt, and *β*-tubulin were purchased from CST (USA); Lycopersion Esculentum Lectin was purchased from Vector Laboratories (USA); DAPI was purchased from Sigma (USA); chemiluminescence detection kit was purchased from Millipore (USA).

### 2.4. Animals

Adult female and male Kunming mice (weighing 25-28 g) were purchased from Beijing Vital River Laboratory Animal Technology Co, Ltd. (Beijing, China; SCXK(J)2016-0011). All experimental protocols were approved by the Animal Care and Use Committee of Shanghai University of Traditional Chinese Medicine (Shanghai, China, PZSHUTCM200703008). The mice were housed in cages separately with controlled temperature and humidity, 12 h light-dark periods, and free access to water and a standard diet. The two estrous cycles were observed by using vaginal smears in female mice before the treatment.

### 2.5. Treatments and Establishment of EID

**Figure 1:**
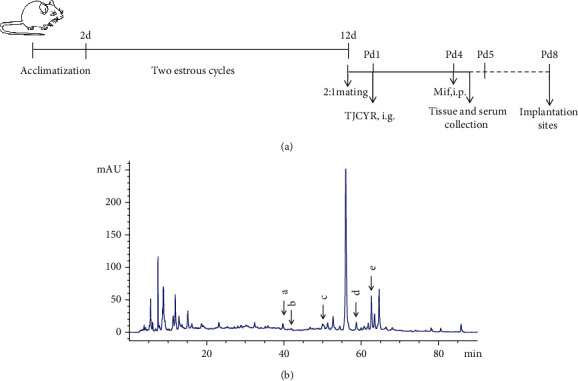
Study scheme and the fingerprinting of TJCYR. (a) Schematic diagram showing the experimental protocol; (b) characteristic fingerprint of TJCYR as analyzed by HPLC. Pd: day of pregnancy; EID: embryo implantation dysfunction; TJCYR: Tiaojing Cuyun Recipe; HPLC: high-performance liquid chromatography; a: Calycosin-7-glucoside; b: Acteoside; c: Salvianolic acid B; d: Icariin; e: Tanshinone IIA.

The female mice were mixed with male mice in a ratio of 2 : 1 to mate overnight in the independent cages. The following morning, the presence of a vaginal plug was considered as an indicator of successful copulation, and this day was classified as day 1 of pregnancy (Pd1). The pregnant mice were then randomly divided into six experimental groups (*n* = 20 in each group), including the control, EID only, progesterone (Prog)+EID, TJCYR-low-dose+EID, TJCYR-medium-dose+EID, and TJCYR-high-dose+EID. In the control and EID-only groups, the mice were given intragastric administration of physiological saline solution once daily for 4 days; the treatment group was given Prog and TJCYR, respectively. Mifepristone (0.1 mg/mouse) was subcutaneously injected at Pd4 in the morning, to establish the EID model [13]. Mice were sacrificed on Pd4 and Pd8, respectively. We obtained blood samples from the orbital vein on Pd4, and the serum was collected to detect hormone levels. The number of implantation sites on Pd8 was recorded. The uteri were harvested on Pd4 to evaluate endometrial receptivity and the potential mechanism. The procedure followed in this experiment is shown in Figure 1(a).

### 2.6. Hematoxylin and Eosin (H&E) Staining

The uteri were dissected and fixed in 4% paraformaldehyde and embedded in paraffin, then serially sectioned at a thickness of 5 *μ*m. Sections were stained with H&E for morphological measurements. The slides were mounted after H&E staining and were examined using an optical microscope (Stemi DV4, Carl Zeiss, Oberkochen, Germany).

### 2.7. Scanning Electron Microscopy (SEM)

The endometria were sliced and fixed in 1.25% (*w*/*v*) glutaraldehyde solution and 1% osmium tetroxide at 4°C for 2 h, respectively. The samples were dehydrated through graded concentrations of ethanol and subsequently dried in a critical-point drier with carbon dioxide, then mounted onto the specimen holder and coated with gold palladium. Finally, all specimens were observed under the SEM (SU8010, Hitachi Instruments, Tokyo, Japan).

### 2.8. Immunofluorescence Staining

Isolated uteri were frozen in optimal cutting temperature compound (OCT) in liquid nitrogen and were serially frozen-sectioned at 8 *μ*m thick. Nonspecific proteins were blocked with 5% fetal bovine serum for 1 h at room temperature (RT). The sections were then incubated with primary antibodies (ER*α*, 1 : 200; ER*β*1, 1 : 500; PR, 1 : 200; Integrin *α*V, 1 : 200; Integrin *β*3, 1 : 100; OPN, 1 : 500; LIF,1 : 500) or fluorescence probe (Lycopersion Esculentum Lectin) overnight at 4°C. The next day, the sections were incubated with the Alexa Fluor 488 nm (green) or Alexa Fluor 555 nm (red) for 1 h at RT in dark, then stained with DAPI (blue). After rinsing three times with PBST, the slides were mounted with Antifade Polyvinylpyrrolidone Mounting Medium and then covered with coverslips. Finally, images were obtained using a Carl Zeiss LSM800 confocal microscope (Carl Zeiss Microscope GmbH, Jena, Germany).

### 2.9. Quantification of NO Content

NO concentration in uterus tissue was measured with the NO assay kit following the user manual. The quantitative determination of nitrite levels represents NO content.

### 2.10. Western Blotting

The uterus tissue was mixed with lysis buffer and the protease inhibitor, then the samples were homogenized and centrifuged, and the supernatants were collected. After determining the protein concentration, the lysates were separated by 10% SDS-PAGE and then transferred to PVDF (0.45 *μ*m, EMD Millipore, Billerica, MA, USA). The membranes were blocked with 5% nonfat milk for 1 h at RT and then probed overnight at 4°C with primary antibodies ER*α* (1 : 1000), ER*β*1 (1 : 1000), PR (1 : 1000), Integrin *α*V (1 : 1000), Integrin *β*3 (1 : 1000), OPN (1 : 500), LIF (1 : 1000,), VEGF (1 : 1000), Akt (1 : 1000), p-Akt (1 : 1000), eNOS (1 : 1000), p-eNOS (1 : 1000), GAPDH (1 : 3000), or *β*-tubulin (1 : 1000). Next, the membranes were washed and incubated with the appropriate secondary antibody for 1 h at RT. Finally, signals were detected using a chemiluminescence detection kit (EMD Millipore, Billerica, MA, USA) and the FluorChem E imaging system (ProteinSimple, San Francisco, CA, USA); the protein band densities were quantified by using an image analysis system (Alpha View SA, ProteinSimple, San Francisco, CA, USA) and expressed as ratios to GAPDH or *β*-tubulin.

### 2.11. Statistical Analysis

All results were expressed as means ± SEM. Differences were compared by one-way analysis of variance (ANOVA) and were considered significant at *P* < 0.05. All statistical tests were performed with GraphPad Prism software version 5.0.

## 3. Results

### 3.1. Quality Evaluation of TJCYR by HPLC

According to the Pharmacopoeia of the People's Republic of China (2020 version), the indicative quality control components of the TJCYR are Calycosin-7-glucoside (standard substance for *Astragalus membranaceus* (Fisch.) Bunge), Acteoside (standard substance for *Rehmannia glutinosa (*Gaert*.)* Libosch.ex Fisch.et Mey.), Salvianolic acid B (standard substance for *Salvia miltiorrhiza* Bge.), Icariin (standard substance for *Epimedium brevicornu* Maxim.), and Tanshinone IIA (standard substance for *Salvia miltiorrhiza* Bge.), respectively. In this study, we established a method for determination of multiple indicator compounds by HPLC, which can effectively determine multiple indicator components in the compound at the same time. The results are shown in Figure 1(b). HPLC chromatographic results showed that the indicator components of these TCM components in the recipe were effectively transferred to the extract during water decocting. That is, TJCYR could play efficacy in the mice treated with TJCYR by intragastric administration. It well guaranteed the quality and pharmacodynamics of TJCYR.

### 3.2. TJCYR Enhancement of Blastocyst Implantation

**Figure 2:**
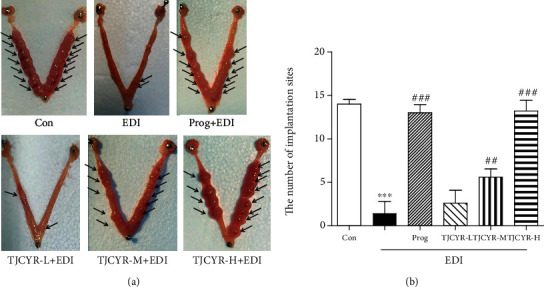
TJCYR increased the number of implantation sites. (a) A representative photograph showing the number of implantation sites (arrows) at Pd8; (b) quantification of implantation sites (*n* = 8). Results are expressed as mean ± SEM. ^∗∗∗^*P* < 0.001 versus control; ^##^*P* < 0.05 and ^###^*P* < 0.01 versus EID only. EID: embryo implantation dysfunction; Prog: progesterone; TJCYR: Tiaojing Cuyun Recipe; TJCYR-H: high-dose TJCYR; TJCYR-M: medium-dose TJCYR; TJCYR-L: low-dose TJCYR.

The numbers of blastocyst sites were recorded on Pd8. As indicated in Figure 2, the embryos appear well-developed and show a string-of-beads arrangement in the control group, while the embryos were markedly tiny and runtish in the EID group, which suggested that the EID was a successful model. Treatment with TJCYR increased the implantation numbers and profoundly promoted the development and distribution of the embryos, especially in mice administered with TJCYR-H, so this treatment was used in the following study.

### 3.3. TJCYR Improved EID-Induced Endometrium Morphological Changes

**Figure 3:**
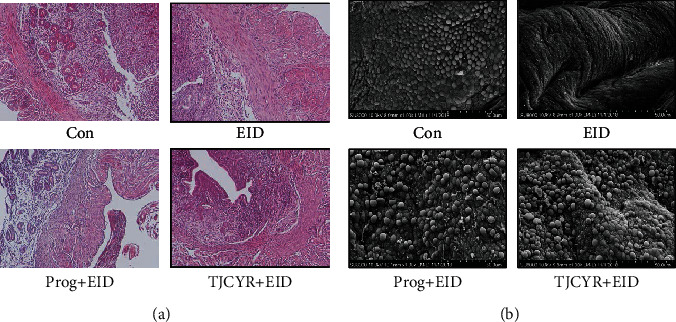
Effect of TJCYR on EID-induced changes in endometrial morphology. (a) H&E showing pathological changes in the endometrium (×200, *n* = 2); (b) SEM showing ultrastructure changes in pinopodes (*n* = 2).

For HE and SEM, all samples were assigned a random number to minimize technical and investigator bias by a double-blinded way. The HE and SEM were read by the professional of pathology and electron microscope in Science and Technology Experiment Center of Shanghai University of Traditional Chinese Medicine, respectively. And then, data was analyzed to reveal the result of morphological changes in every group. As shown in Figure 3(a), H&E staining was used to evaluate the pathological changes of the endometrium. In the EID-only group, loose endometrial stromal tissue and insufficient glands and vessels were found, in contrast to the mice treated with TJCYR. To further assess the morphological TJCYR-related changes on EID, we investigated the effect of TJCYR on the pinopodes using SEM. As shown in Figure 3(b), in the control group, the majority of well-developed pinopodes were evenly distributed over the endometrial epithelial surfaces, while on EID-induced surfaces, well-developed pinopodes were sparse and a few developing pinopodes were seen. However, the pinopodes in this group improved significantly following treatment with TJCYR. The results suggest that TJCYR can improve the endometrial morphology and vasculature in an EID model, with benefits for embryo implantation.

### 3.4. The Effect of TJCYR on Hormones and Hormone Receptors

**Figure 4:**
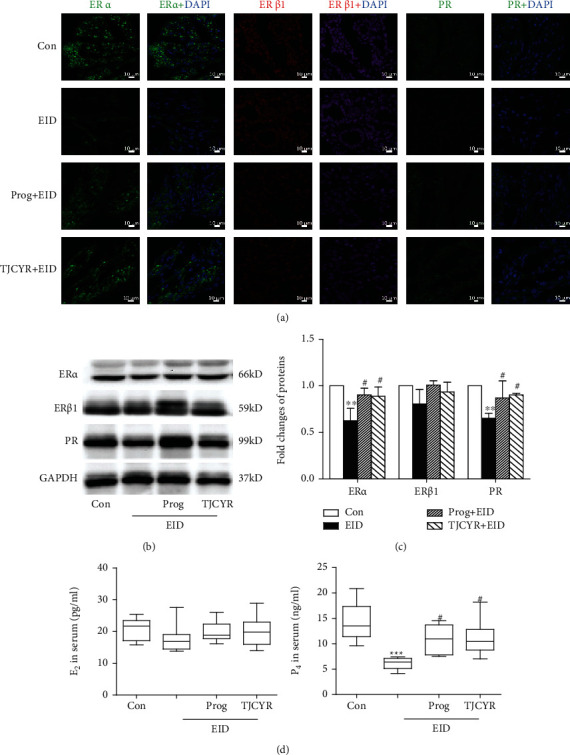
Effect of TJCYR on EID-induced changes in the hormones and receptors. (a) Representative confocal fluorescent images of the uterine sample with ER*α* (green), ER*β*1 (red), and PR (green), respectively (×20, *n* = 2). (b) Protein levels of ER*α*, ER*β*1, and PR in uterine tissue were determined by Western blotting (*n* = 3); (c) quantification of protein levels. (d) The serum levels of E_2_ and P_4_ were tested by radioimmunoassay. Results are expressed as mean ± SEM. ^∗∗∗^*P* < 0.001 and ^∗∗^*P* < 0.01 versus control; ^#^*P* < 0.05 versus EID only. EID: embryo implantation dysfunction; Prog: progesterone; TJCYR: Tiaojing Cuyun Recipe; E_2_: 17*β*-estradiol; P_4_: progesterone; ER*α*: estrogen receptor alpha; ER*β*1: estrogen receptor beta1; PR: progesterone receptor.

The receptive mouse uterus displays stromal proliferation and epithelial differentiation, indicating readiness for blastocyst implantation, and is governed by ovarian hormones, such as 17*β*-estradiol (E_2_) and progesterone (P_4_). We tested the serum content of E_2_ and P_4_ in the serum. For the hormonal analysis, all samples were also randomly assigned; the test of hormone was conducted by Shanghai Xinfan Biological Technology Co., Ltd. And the results were analyzed in a double-blind way. We found similar E_2_ level in the EID only and control groups, but the level of P_4_ was notably reduced in the EID only group. Treatment with TJCYR may inhibit the P_4_ decrease induced by EID. The primary mediators of these E_2_- and P_4_-induced events are their receptors, estrogen receptor (ER) and progesterone receptor (PR). We also test the expression of ER and PR by immunofluorescence (IF) and Western blot (WB). ER include ER-alpha (ER*α*) and ER-beta1 (ER*β*1). The result of IF showed that the fluorescence intensity of ER*α* in the EID-only group was slightly weaker than in the controls, and no difference was found in the fluorescence intensity of ER*β*1 between the EID-only group and the control group. However, the fluorescence intensity of PR was clearly weaker in the EID-only group than that in the control group, and treatment with TJCYR could enhance the expression of PR. In the WB, we also found that PR protein was clearly decreased in the EID-only group and that TJCYR could reverse that change. These results show that the changes in P_4_ and PR were clearly apparent in the EID model and that treatment with TJCYR could regulate P_4_ and PR (Figure 4).

### 3.5. The Treatment of TJCYR Increases Endometrial Receptivity-Related Markers

**Figure 5:**
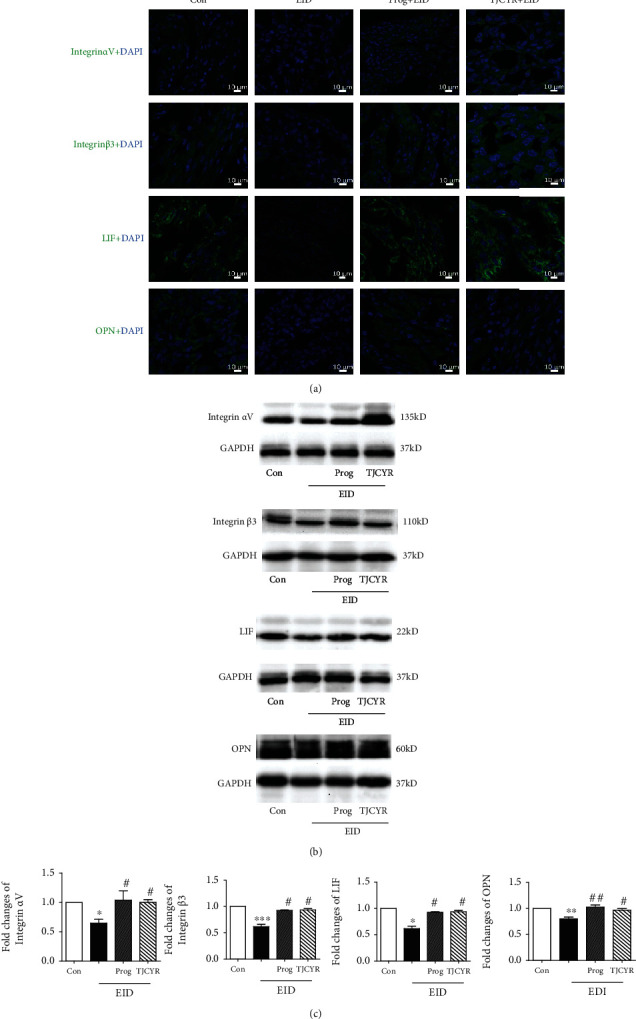
Effect of TJCYR on the biomarkers of endometrial receptivity. (a) Representative confocal fluorescent images of the uterine sample with Integrin *α*V, Integrin *β*3, LIF, and OPN, respectively (×20, *n* = 2). (b) Protein levels of Integrin *α*V, Integrin *β*3, LIF, and OPN in uterine tissue were determined by Western blotting (*n* = 3); (c) Quantification of protein levels. Results are expressed as mean ± SEM. ^∗^*P* < 0.05, ^∗∗^*P* < 0.01, and ^∗∗∗^*P* < 0.001 versus control; ^#^*P* < 0.05 and ^##^*P* < 0.01 versus EID only. EID: embryo implantation dysfunction; Prog: progesterone; TJCYR: Tiaojing Cuyun Recipe.

Several molecular markers are related to endometrial receptivity, including Integrin *α*V, Integrin *β*3, LIF, and OPN. As shown in Figure 5, immunofluorescence and Western blot analysis showed that the expression of Integrin *α*V, Integrin *β*3, LIF, and OPN significantly decreased in the EID-only group. TJCYR was found to attenuate the EID-induced damage by increasing the expression of Integrin *α*V, Integrin *β*3, LIF, and OPN. These results are consistent with the changes found in pinopodes as shown in Figure 3(b).

### 3.6. TJCYR Treatment Promotes Endometrial Angiogenesis

**Figure 6:**
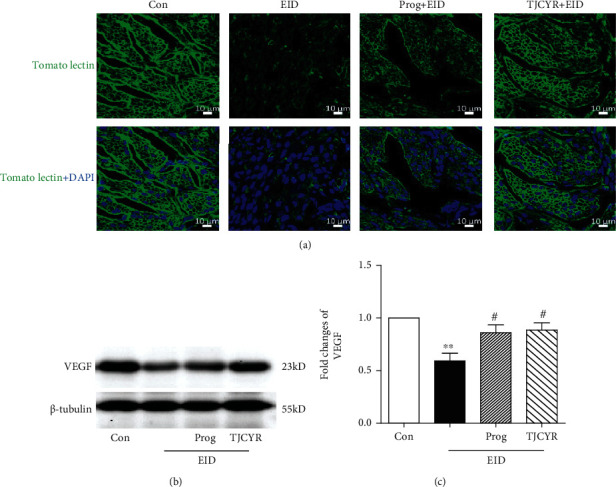
Effect of TJCYR on angiogenesis and hypoxia. (a) Representative confocal fluorescent images of the uterine sample with Tomato Lectin (green) and DAPI (blue) (×20, *n* = 2); (b) protein levels of VEGF in uterine tissue were determined by Western blotting (*n* = 3); (c) quantification of protein levels. Results are expressed as mean ± SEM; ^∗^*P* < 0.05 and ^∗∗^*P* < 0.01 versus control; ^#^*P* < 0.05 versus EID only. EID: embryo implantation dysfunction; Prog: progesterone; TJCYR: Tiaojing Cuyun Recipe.

Angiogenesis is one of the important biological events which may be involved in implantation. Tomato Lectin is recognized as the most sensitive vessel marker, the fluorescence intensity of which may reflect vascular density. As shown in Figure 6(a), high immunofluorescence was detected using Tomato Lectin in the control and TJCYR treatment groups, while the immunofluorescence appeared weak in the EID only group. In contrast, microvascular density was dramatically enhanced in the TJCYR group. VEGF is an important mediator of angiogenesis with beneficial effects on endometrial receptivity and plays a key role in the embryonic development of mice. As shown in Figures 6(b) and 6(c), treatment with TJCYR clearly inhibited the decrease of VEGF in mice subjected to EID, which is consistent with the immunofluorescence analysis (Figure 6(a)) which showed increased vascular density.

### 3.7. TJCYR Improved Angiogenesis through the PI3K/Akt/eNOS Signaling Pathway

**Figure 7:**
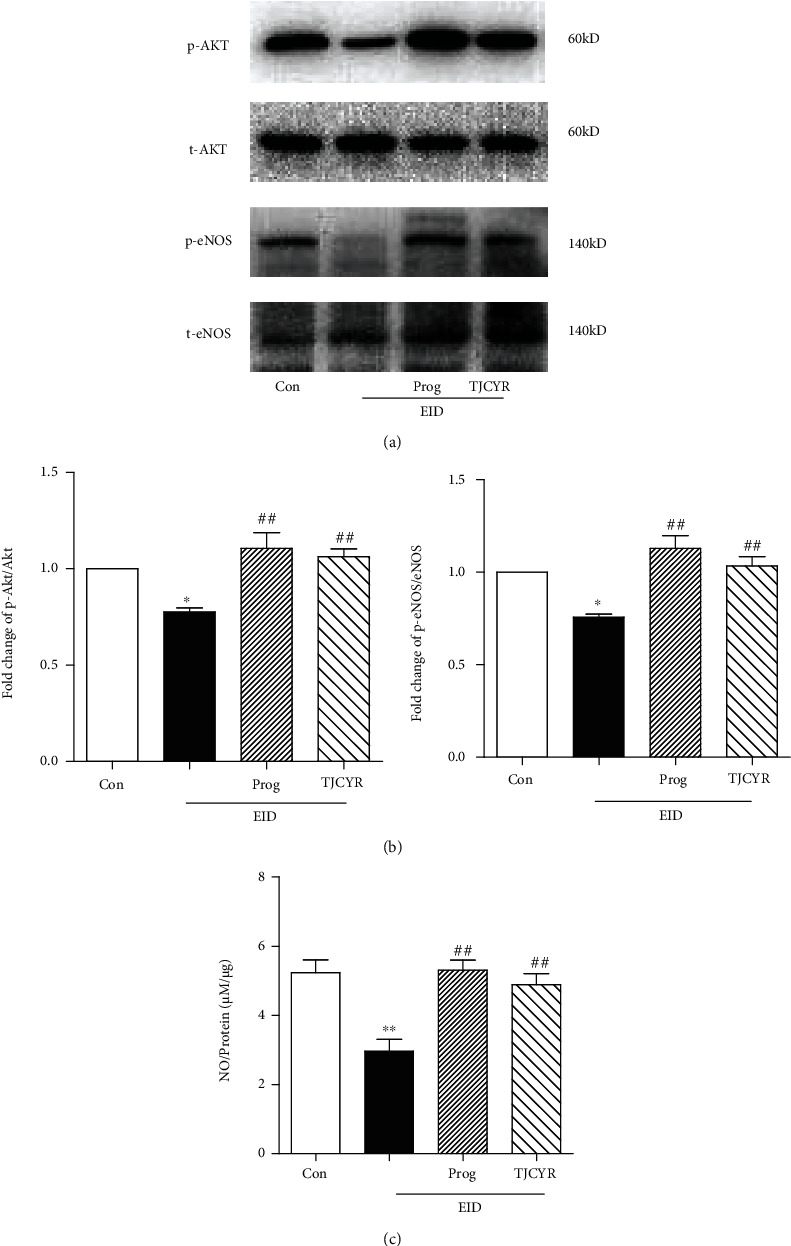
Effect of TJCYR on the PI3K/Akt/eNOS signaling pathway. (a) Protein levels of the PI3K/Akt/eNOS signaling pathway in uterine tissue were determined by Western blotting (*n* = 3); (b) quantification of protein levels. Results are expressed as mean ± SEM; (c) NO production was shown by NO/protein (*n* = 10). Results are expressed as mean ± SEM. ^∗^*P* < 0.05 and ^∗∗^*P* < 0.01 versus control; ^##^*P* < 0.01 versus EID only. EID: embryo implantation dysfunction; Prog: progesterone; TJCYR: Tiaojing Cuyun Recipe; NO: nitric oxide.

The PI3K/Akt/eNOS pathway is downstream of VEGF, which participates in angiogenesis. Here, we found that the expressions of p-Akt and p-eNOS were all reduced in the EID-only group compared to the control group, accompanied by a decline in NO. All of these decreased expressions were restored by treatment with TJCYR (Figure 7). These data suggest that TJCYR stimulated the PI3K/Akt/eNOS signaling pathway, which was deactivated by EID.

## 4. Discussion

Successful embryo implantation is a highly orchestrated process involving blastocyst-uterine interactions. While blastocyst quality has been extensively studied, endometrial receptivity is equally important. Endometrial receptivity is defined as “that period of endometrial maturation during which the trophectoderm of the blastocyst can attach to the endometrial epithelial cells and subsequently proceed to invade the endometrial stroma and vasculature” [14]. Therefore, improving endometrial receptivity is key to raising the pregnancy rate. The pinopodes have been considered as the characteristic morphologic markers of endometrial receptivity and of the implantation window [13, 15]. The pinopodes are membrane protrusions on the apical surface of luminal epithelium on Pd4 in mice. They prevent cilia from sweeping the blastocyst away as well as promoting withdrawal of uterine fluid and closure of the uterine cavity [4, 16]. In our study, the specimens were examined by SEM to detect pinopodes. The results revealed that TJCYR may enhance the number of fully developed pinopodes, accompanied by reduced distribution of cilia, which may provide nutrients for the embryo and enable its attachment to the uterine endometrium. The pinopodes play a key role in the initial stage of implantation by promoting attachment of the embryo.

The endometrium is the most direct target organ for estrogen and progesterone, among which E2 and P4 are the most important regulatory factors. The formation of pinopodes depends on the influence of estrogen and progesterone, the physiological effects of which depend on the mediation of estrogen receptors and progesterone receptors in endometrial glandular epithelial cells [17]. Therefore, estrogen, progesterone, and their receptors are important factors for the formation of good endometrial receptivity. Some studies [18, 19] have shown that the establishment of endometrial receptivity requires sufficient estrogen stimulation before ovulation and the supportive effect of progesterone on the endometrium after ovulation. In this study, we described the protective effects of TJCYR against EID, and we found that progesterone and its receptor were increased notably with TJCYR treatment.

Pregnancy is a versatile and dynamic process for the implanting embryo, requiring a series of rather complicated and synchronous morphological and biochemical changes. The appearance of pinopodes is consistent with the expression of several molecular markers of endometrial receptivity, including Integrin, leukemia inhibitory factor (LIF), and osteopontin (OPN). It is meaningful to analyze pinopodes and the molecular biomarkers together, hence our further studies exploring the effect of TJCYR on the expression of Integrin, LIF, and OPN. Integrins are cell surface receptors that are involved in cell-to-cell and extracellular matrix adhesion. Some Integrins increase during the implantation window. There are many isoforms of Integrins in mammals, but only three (*α*1*β*1, *α*4*β*1, and *α*V*β*3) have been found to have roles in implantation, *α*V*β*3 playing the most conspicuous role [16]. Integrin *α*V*β*3 is a potential receptor for blastocyst attachment and is localized on pinopodes [16]. Some studies have revealed that blockage of Integrin *α*V*β*3 or lack of Integrin *β*3 may be related to unexplained infertility [20]. LIF is a member of the interleukin-6 family of cytokines, which plays a critical role in implantation. Pinopodes release secretory vesicles containing LIF in the uterine lumen to enable trophoblast invasion and affecting immune tolerance during implantation [16, 21]. Some studies [22] have shown that the deletion or mutation of LIF induced implantation failure. Other studies [23, 24] reported that endometrial LIF and receptor were higher around the time of implantation in fertile women compared with women with unexplained infertility. Other findings [25] have shown that LIF may maintain the proper development of the endometrium and implantation receptivity by regulating downstream target genes. OPN is one of the cofactors involved in cell adhesion and invasion during the implantation process, is an acidic member of the small integrin-binding ligand family of proteins [26], and has been shown to be maximally expressed in the epithelial layer in human, mouse, and rabbit uterine. OPN is therefore an important constituent of the uterus during pregnancy [26, 27]. In this study, the EID group showed histopathological lesion characteristic of pinopodes, as well as low expression of Integrin *α*V, Integrin *β*3, LIF, and OPN, while the expression of these biochemical markers was significantly increased in mice treated with TJCYR. These data strongly demonstrate the benefits of TJCYR in endometrial receptivity.

During the implantation window, a rich vascular network is necessary to supply nutrients and oxygen. As is well known, oxygen is abundant where blood supply is rich [28]. In clinical practice, blood flow scanning is the current and preferred approach for assessing endometrial function [29]. It is well established that proper endometrial vascular development and maintenance are crucial for successful pregnancy [30]. Insufficient angiogenesis may result in poor endometrial receptivity [31]. Based on Lectin immunostaining showing higher vascular density in TJCYR treatment, we next explored whether VEGF expression is increased with this treatment. VEGF has a predominant role in successful implantation and maintenance of pregnancy by increasing vascular permeability or forming vascular networks. It has been reported that VEGF knockout mice do not produce viable offspring [32]. The expression of VEGF increases significantly during implantation windows, which suggests that it promotes angiogenesis and the establishment of capillary networks, further improving endometrial receptivity and promoting embryo implantation [33]. Alternatively, deficiency of VEGF may induce the reduction of angiogenesis at the implantation site and lead to miscarriage [34]. VEGF inhibitors are used to achieve contraception [35]. In this study, we found a poor vascular network in the mice treated with EID only; that treatment with TJCYR significantly inhibited the decline in vessel density and expression of VEGF in the EID-only group and improved endometrial blood circulation. All the data showed that VEGF regulated angiogenesis and built endometrial microenvironment for embryo implantation.

It is well known that, as the downstream signaling pathway of VEGF, the activated PI3K/Akt pathway regulates cell proliferation, differentiation, apoptosis, cell cycle, protein synthesis, cell energy metabolism, and other functions [36, 37]. It had been demonstrated that the role of the PI3K/Akt pathway may vary completely with conditions. In this study, we found that after the PI3K/Akt signaling pathway was activated by increased VEGF, the eNOS was sequentially activated. eNOS acts as a key regulator of angiogenesis by mediating the speed of NO enzyme generation. NO is an important angiogenic substance produced by healthy endothelial cells to support vascular homeostasis and blood flow. During the reproductive processes, NO plays a critical role in maintaining blood vessel stability at the implantation site, further safeguarding embryo implantation and pregnancy maintenance. In the present study, we found that the expressions of p-Akt and p-eNOS were all reduced in the EID mice compared to the control group. Conversely, treatment with TJCYR markedly increased the expression of VEGF and subsequently increased the activation of the PI3K/Akt/eNOS pathway.

## 5. Conclusion

The results of the present study revealed that TJCYR can activate the PI3K/AKT/eNOS signaling pathway to improve endometrial microcirculation and blood flow, enhancing endometrial receptivity and embryo implantation, which were correlated with the characteristic changes in endometrial morphology and the upregulation of molecular markers for endometrial receptivity.

## Figures and Tables

**Table 1:** Tiao Jing Cu Yun Recipe (TJCYR) components.

Chinese term	Generic name	Scientific name	Weight (g)	Product lot
Dangshen	*Codonopsis pilosula* (Franch.) Nannf.	Codonopsis radix	20	171011
Danshen	*Salvia miltiorrhiza* Bge.	Salviae miltiorrhizae radix et rhizoma	20	170701
Danggui	*Angelica sinensis* (Oliv.) Diels	Angelicae sinensis radix	20	170606
Huangqi	*Astragalus membranaceus* (Fisch.) Bunge.	Astragali radix	20	160829
Shudihuang	*Rehmannia glutinosa* (Gaert.) Libosch.ex Fisch.et Mey.	Rehmanniae radix praeparata	15	170420
Bajitian	*Morinda officinalis* How.	Morindae officinalis radix	12	171219
Yinyanghuo	*Epimedium brevicornu* Maxim.	Epimedii folium	12	170723

## Data Availability

The data are available upon direct request to the corresponding author.
